# Imbalance in PB IL-17-Secreting and Regulatory Cells in Pars Planitis Is Associated with Dysregulation of IFN-*γ*-Secreting Cells, Especially in Patients with Clinical Complications

**DOI:** 10.1155/2020/9175083

**Published:** 2020-07-31

**Authors:** Agata Kosmaczewska, Joanna Przeździecka-Dołyk, Anna Turno-Kręcicka, Lidia Ciszak, Aleksandra Szteblich, Agnieszka Węgrzyn, Irena Frydecka, Marta Misiuk-Hojło

**Affiliations:** ^1^Hirszfeld Institute of Immunology and Experimental Therapy, Polish Academy of Sciences, Wroclaw, Poland; ^2^Department and Clinic of Ophthalmology, Faculty of Postgraduate Medicine, Medical University of Wroclaw, Wroclaw, Poland; ^3^Department of Optics and Photonics, Faculty of Fundamental Problems of Technology, Wroclaw University of Science and Technology, Wroclaw, Poland; ^4^Deanery of Clinical Sciences, College of Medicine and Veterinary Medicine, University of Edinburgh, Edinburgh, UK; ^5^Łukasiewicz Research Network - PORT Polish Center for Technology Development, Wroclaw, Poland

## Abstract

**Results:**

In patients, an increase in the population of Th17-secreting cells negatively correlated with the abundance of both IFN-*γ*-producing and T regulatory as well as suppressor cells, regarding all the phenotypes studied. Although a strong dependence of the PB Th1 cell compartment on the duration of the disease was observed, it was limited to the subgroup of patients with macular edema only. The frequency of B regulatory cells was unchanged compared to controls.

**Conclusions:**

In pars planitis, the alterations in lymphocyte cell distribution affect primarily the T cell repertoire. The imbalance in PB Th1/Th17/Treg cells creates proinflammatory conditions, strengthening the suggestion that the immune background may play a role in pars planitis pathogenesis. Also, circulating Th1 level may be of potential clinical relevance in terms of prediction of a more severe course of the disease.

## 1. Introduction

According to the Standardization of Uveitis Nomenclature (SUN), pars planitis is an intermediate uveitis of unknown cause [1]. Pars planitis is characterized by the presence of snowbank and snowball formation, which are chronic inflammatory aggregates, on pars plana and within the vitreous in the absence of an infectious or systemic disease. It is usually considered as a benign form of uveitis, but it may also be complicated by cataract, optic disc edema, vitreous opacities, and cystoid macula edema. The latter is the most common cause of visual morbidity. The incidence of pars planitis is rarely evaluated, and usually, the incidence of intermediate uveitis is assessed; the incidence is approximately 1.5%, whereas the prevalence is 4.0 per 100,000 patients a year (2.8 per active and 5.1 for an inactive form of the disease) [2, 3]. The etiology of pars planitis remains unknown. Only a few articles describe pars planitis as a separate entity (usually, the described group is intermediate uveitis and only single records contribute to pars planitis). From a histological and clinical point of view, the autoimmune etiology of pars planitis is strongly suggestive. In many cases, T helper lymphocytes as predominant cells were detected in the perivascular infiltrates or in snowbanks, suggesting an immune response to some unknown antigen [4, 5]. In fact, snowbanks are formed as an effect of postinflammatory glial proliferation of fibrous astrocytes. Therefore, appropriate control of inflammation in the eye tissue seems to be crucial in order to improve the prognosis of the disease.

There are many controversies with the therapy of pars planitis, including a lack of consensus for the treatment of patients with minimal inflammation. In turn, those with severe tissue injury need an aggressive therapy including corticosteroids, immunosuppressive agents, pars plana vitrectomy, and/or laser photocoagulation [6]. The treatment of pars planitis as an entity of undefined cause in the era of newly introduced biologic treatment into the daily routine appears to bear significant importance in clinical practice. Since biologic treatment with antitumor necrosis factor-alpha (adalimumab) for noninfectious nonanterior uveitis is a standard procedure in common clinical practice, a better understanding of the underlying immunologic changes can contribute to developing better concepts of the therapy. The wide range of biologic treatment available nowadays implies the need for research on diseases that have been considered as idiopathic with an immunological background such as pars planitis.

In the present study, we performed an analysis of the peripheral distribution of pro- (IFN-*γ* and IL-17 secreting) and anti-inflammatory (Treg, Tsup, and Breg) lymphocyte subsets in patients with pars planitis to identify an immune cell population playing a key role in disease pathogenesis. A proven role of certain lymphocyte subpopulations could be useful in determining both the predictor of clinical course and the significance of an appropriate monoclonal antibody used in the treatment. The knowledge on the immunologic background and better understanding of pathomechanisms of pars planitis could be used in terms of the different personalized treatment protocols.

## 2. Material and Methods

### 2.1. Patients

The study population consisted of 15 subjects with PP. The control population comprised 17 healthy blood donors matched for age and sex, with no medical history affecting the immune system such as diabetes, autoimmune diseases (including connective tissue diseases), malignancies, and chronic or acute infections. The project protocol was referred to and approved by the local Bioethics Committee (number: KB-329/2014, acceptance date: 5th June 2014). The trial was performed in compliance with the provisions of the Declaration of Helsinki as well as the guidelines of the International Conference on Harmonisation Good Clinical Practice and local regulations. Blood samples from all participants were collected after informed written consent.

The patients with intermediate uveitis or pars planitis were screened. The inclusion criteria for screening included an age limit of at least 18 years, previously diagnosed intermediate uveitis or pars planitis, and the lack of any oral or local immunosuppressive treatment. According to the Standardization of Uveitis Nomenclature Working Group (SUN) criteria for the diagnosis of pars planitis in all included patients, both the systemic and infectious causes of the uveal manifestation as well as infection were excluded [1]. Every blood sample was tested for *Borrelia burgdorferi*, toxoplasmosis, toxocariasis, *Bartonella pertussis* (levels of IgM and IgG were measured for all above-mentioned factors), QuantiFERON and skin reaction to tuberculin (Mantoux test with tuberculin RT23), and syphilis (Wassermann reaction and VDRL test) as well as for viruses such as HSV-1 and HSV-2, VZV, HHV-7, CMV, Epstein-Barr virus, and HIV (commonly used tests). An X-ray examination of the chest and rheumatological and neurological consultation with indicated appropriate tests were performed in order to rule out any systemic disease. The inclusion criteria included an age limit of at least 18 years, confirmed diagnosis of pars planitis, and negative test results for infectious causes and systemic diseases. The full inclusion and exclusion criteria can be found in Table 1. For all included patients, both eyes were eligible for participation in the study. A clinical summary of additional tests is presented in Tables S1 and S2 (see Supplementary Materials).

### 2.2. Ophthalmological Evaluation

Patients included in the trial underwent a full ophthalmological evaluation including best-corrected visual acuity for distance (BCVA) and for near vision (NV), intraocular pressure (IOP) measured by Goldmann applanation tonometry, slit-lamp evaluation of the anterior segment with the assessment of the inflammation (according to the SUN criteria for anterior chamber cell count and flare, a higher score indicates that more cells/fibrins are visible in the anterior chamber), assessment of vitreous haze grade (according to the National Eye Institute (NEI) criteria adapted by the SUN, a higher score indicates greater severity of pars planitis), ophthalmoscopy (with fundus photography), and spectral-domain optical coherence tomography (SD-OCT) of the macula along with the evaluation of morphology and thickness of the submacular choroid (with enhanced deep imaging—EDI) with the SD-OCT Spectralis system. A summary of performed ophthalmological examinations including grading scales and equipment specification used is provided in Table S3 (see Supplementary Materials).

Additionally, the patients were asked to fill in the NEI Visual Functioning Questionnaire-25 (VFQ-25). The test was translated to a national language and accepted as a tool by the local Bioethics Committee.

### 2.3. Cell Preparation and Cytometric Analysis

Peripheral blood mononuclear cells (PBMCs) were isolated by buoyant density-gradient centrifugation on Lymphoflot (Biotest, Germany) from 40 ml of freshly collected peripheral venous blood (PB). The peripheral blood mononuclear cells were stained with several combinations of antihuman fluorochrome-conjugated monoclonal antibodies (mAbs) for three-color analysis.

Percentages of cytokine-producing T cells (Th1 and Th17) were calculated after stimulation with 25 ng/ml of phorbol 12-myristate 23-acetate (PMA, Sigma-Aldrich) and 1 *μ*g/ml of ionomycin (Ion) (Sigma-Aldrich) in the presence of 10 *μ*g/ml of brefeldin A (BFA, protein transport inhibitor) (Sigma-Aldrich) for 4 h at 37°C in a humidified atmosphere containing 5% CO_2_. Because incubation with PMA triggers internalization and degradation of the CD4 molecule, which would affect the identification of Th1 (phenotyped as CD4+IFN-*γ*+) as well as Th17 cells (characterized as CD4+IL-17+) [7, 8], both subpopulations were identified as CD3+CD8-IFN-*γ*+ and CCR4-CXCR3+IFN-*γ*+ as well as CD3+CD8-IL-17+ and CCR4+CCR6+IL-17+ cells, respectively. Detection of Th1 and Th17 cells within the CD3+CD8- subset was in line with previous recommendations [7, 9] and in accordance with experimental protocols applied in numerous studies [10–12]. Given that CCR4 was found to be expressed mainly on Th2 and Th17 cells [13, 14], and Th17 cells display CCR6 as a phenotypical marker [15], we also defined CCR4+CCR6+IL17+ cells as the Th17 subset. Similarly, having reported that CCR4 is not substantially expressed by Th1, NK cells, or B cells [13], and CXCR3 is thought to be a marker for identification of Th1 cells [16, 17], we defined CCR4-CXCR3+IFN-*γ*+ cells as the Th1 subset, as well.

Directly after PMA+Ion stimulation, PBMCs were surface-stained with respective mAbs as follows: anti-CD3/PerCP (BD Biosciences), anti-CD8/PE (BD Biosciences), anti-CCR4/PerCP, anti-CXCR3/PE (BioLegend), and anti-CCR6/PE (BioLegend). Then, after fixation and permeabilization with Fixation/Permeabilization Buffer Set (eBioscience), the cells were incubated with anti-IFN-*γ*/FITC (BD Biosciences) or anti-IL-17/FITC (BioLegend) mAbs for 30 min in the dark, at room temperature.

For analysis of the suppressor cell subpopulations, including CD4+CD25hiCD127- Treg cells, CD19+CD24hiCD38hi Breg cells, and CD8+CD28-FOXP3+ Tsup cells, PBMCs were aliquoted into tubes directly after isolation for further staining with the following mAbs: anti-CD4/PerCP (BD Biosciences), anti-CD25/FITC (BD Biosciences), anti-CD127/PE (BioLegend), anti-CD19/FITC (BD Biosciences), anti-CD24/PerCP (BioLegend), anti-CD38/PE (BD Biosciences), anti-CD8/PerCP (BD Biosciences), and anti-CD28/FITC (BD Biosciences), respectively. Then, for intracellular staining, the cells were fixed and permeabilized with Fixation/Permeabilisation Buffer Set (eBioscience) according to the manufacturer's instructions following subsequent incubation with anti-human FOXP3/PE (BD Biosciences) mAbs for 30 min in the dark, at room temperature.

Directly after immunostaining, the cells were washed and analyzed by flow cytometry using a FACScan cytometer (Becton Dickinson) equipped with CellQuest software (BD Biosciences). In each case, appropriate fluorochrome-labeled isotypic controls were used for gate settings. A total of 30,000 events were recorded for each sample before any electronic gate setting.

### 2.4. Statistical Analysis

Statistical analysis was performed using the package Statistica 7.1. The Mann-Whitney *U* and Kolmogorov-Smirnov tests were used to identify a statistically significant difference (*p* < 0.05) for the primary hypothesis of a difference in lymphocyte subpopulations between the pars planitis group (PP) and the control group (C). The secondary outcomes, defined as the evaluation of the differences in the proportion of lymphocyte subpopulations in patients suffering from pars planitis with or without cooccurring macular edema (MEG for macular edema subgroup; WMEG for subgroup without macular edema), were considered as statistically significant for *p* < 0.025. The relationship between the analyzed variables was tested with Spearman's rank correlation coefficient.

## 3. Results

To assess the immunological status of untreated subjects with PP in comparison with the control group, the frequencies of immune cell subsets in PB were examined. Our attention was focused on the effector IFN-*γ*- (Th1) and IL-17-producing (Th17) as well as suppressor (Treg, Tsup, and Breg) lymphocytes, since these cell populations have been suggested to be implicated in the pathogenesis of uveitis [18]. Such studies in pars planitis are still lacking so far.

### 3.1. Increased Th17/Th1 Ratio in Pars Planitis

**Figure 1:**
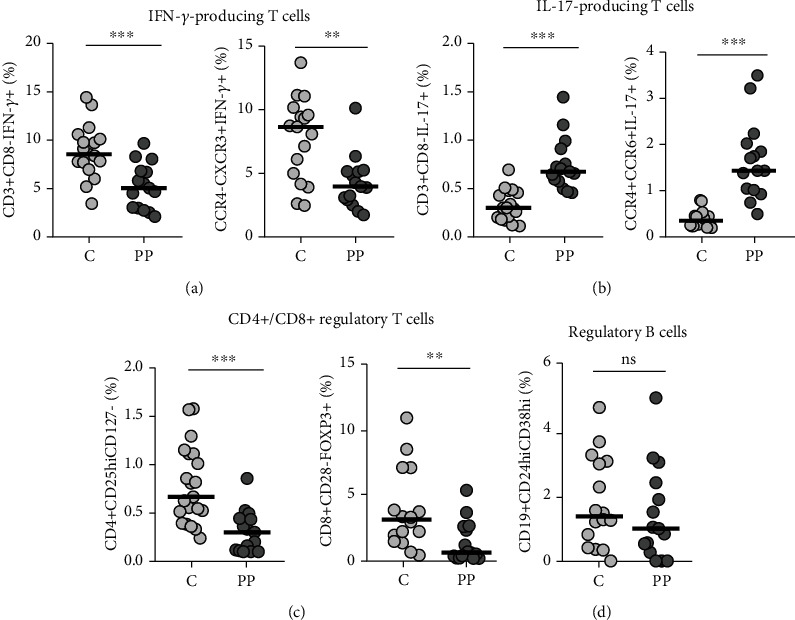
Distribution of IFN- and IL-17-secreting cells in PB of pars planitis patients (PP) and healthy controls. Subjects with PP are characterized by decreased frequency of IFN-*γ*-producing T cells (a), increased frequency of IL-17-producing cells (b), and reduced frequency of both regulatory CD4+ and CD8+ T cells (c, d). There was no statistically significant difference in frequency of Breg cells between groups. C: healthy control group; PP: pars planitis. ^∗^*p* < 0.05, ^∗∗^*p* < 0.01, and ^∗∗∗^*p* < 0.001.

Regarding the abundance of IFN-*γ*- and IL-17-secreting T cells in circulation, there were statistically significant differences between PP patients and healthy donors. The values of Th1 and Th17 cells are shown in Figure 1(a), Figure S1 (see Supplementary Materials), and Figure 1(b), Figure S2 (see Supplementary Materials), respectively.

In detail, markedly decreased frequencies of both CD3+CD8-IFN-*γ*+ and CCR4-CXCR3+IFN-*γ*+ cells in patients with PP compared to the corresponding cells from the control group were observed (*p* < 0.001 and *p* < 0.01, respectively). In contrast, a subset of IL-17-producing cells, described as CD3+CD8-IL-17+ and CCR4+CCR6+IL-17+, was significantly larger in PP patients than in controls (both *p* < 0.001).

### 3.2. Downregulation of Regulatory T Cell Subsets in Pars Planitis

Regulatory and suppressor T cell subsets were detected within both CD4+ and CD8+ T lymphocytes and identified, respectively, as CD4+CD25hiCD127- and CD8+CD28-FOXP3+ cells. The frequencies of these cells in PB of patients and controls are shown in Figure 1(c) and in the Supplementary Figure S3A, S3B.

We found a significant decrease in the proportion of both CD4+CD25hiCD127- T regulatory cells (Treg) and CD8+CD28-FOXP3+ T suppressor cells (Tsup) in patients in comparison with healthy subjects (*p* < 0.001 and *p* < 0.01, respectively).

### 3.3. Nonaffected Regulatory B Cell Subset in Pars Planitis

Whereas lower abundance of regulatory B cells (Breg) determined as CD19+CD24hiCD38hi cells was observed in the circulation of PP patients compared to the control group, the difference was not statistically significant (*p* > 0.05). The B cell abundance is presented in Figure 1(c) and Supplementary Figure S3C.

### 3.4. Increase in Th17 Subset Is Associated with Both Treg and Th1 Downregulation in Pars Planitis

**Figure 2:**
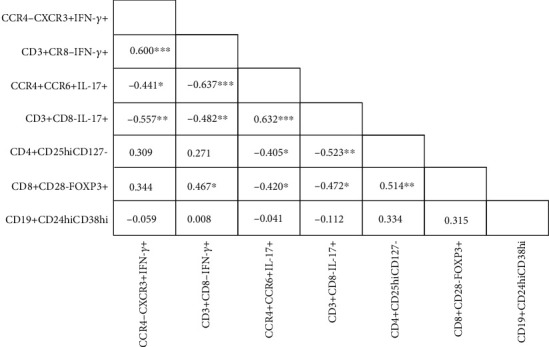
Correlations between effector and regulatory cell subpopulations in pars planitis (PP) patients. Frequency of IL-17-producing cells negatively correlates with the frequency of both IFN-*γ*-producing cells and regulatory/suppressor T cells. ^∗^*p* < 0.05, ^∗∗^*p* < 0.01, and ^∗∗∗^*p* < 0.001.

By analyzing the correlations in the patient group, we examined whether any relationships between the frequency of certain subsets of effector and/or regulatory cell subpopulations exist. The obtained results are summarized in Figure 2.

It was found that the proportion of peripheral IL-17-producing cells within either CD3+CD8- or CCR4-CXCR3+ subsets was negatively correlated with the frequency of both CD4+CD25hiCD127- and CD8+CD28-FOXP3+ regulatory cells (*p* < 0.01). Similarly, all associations between IL-17- and IFN-*γ*-producing cells were negative as regards each cell phenotype studied (*p* < 0.01). There was no marked correlation of Breg cells with other immune cells (*p* > 0.05).

### 3.5. Association of Th1 Subsets with Duration of Severe Pars Planitis

**Figure 3:**
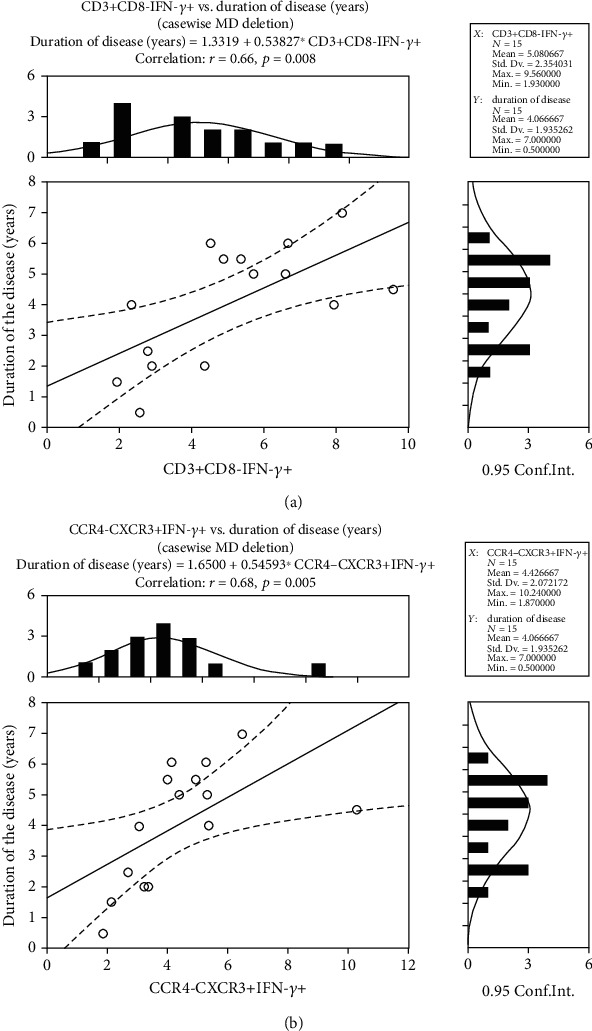
Correlation of disease duration with Th1 cells phenotyped as (a) CD3+CD8-IFN-*γ*+ and (b) CCR4-CXCR3+IFN-*γ*+ cells.

Among examined effector and regulatory cell subsets and clinical characteristics, we found only the correlation of PB Th1 cell subsets, detected as CD3+CD8-IFN-*γ*+ and CCR4-CXCR3+IFN-*γ*+, with a duration of pars planitis (*r* = 0.65, *p* = 0.008 and *r* = 0.68, *p* = 0.004, respectively) (Figure 3). However, further detailed analysis relying on the subdivision of our cohort of PP patients into the MEG and WMEG subgroups revealed, in fact, no relationships of CD3+CD8-IFN-*γ*+ and CCR4-CXCR3+IFN-*γ*+ subsets with PP duration in the WMEG subgroup (*p* > 0.05), whereas such a correlation in the MEG subgroup turned out to be even stronger, regarding both respective phenotypes of Th1 cells (*r* = 0.81, *p* = 0.027 and *r* = 0.77, *p* = 0.041, respectively).

## 4. Discussion

Our paper describes the immunologic pattern of subjects suffering from pars planitis in comparison to the healthy control group. It has been assumed that immune alterations in the circulation may result from or even reflect changes observed in the inflamed tissues, thus giving us the opportunity to assess the local disorders. Under physiologic conditions, the retinal pigment epithelium (RPE) of the eye exhibits a strong immunoregulatory function by downregulation of Th1, Th17, Th22, B cells, CD8+ T cells, macrophages, and dendritic cells, while upregulating T regulatory and suppressor myeloid cells [18]. Therefore, the RPE has a major capacity for shifting the local balance towards immune tolerance, whereby an eye is thought to be an immune-privileged organ. The important role of RPE in the creation of the immune-privileged microenvironment in the ocular tissues has been summarized previously [19]. Recent reports also suggest that its breakdown may play a key role in the pathogenesis of pars planitis, which is an idiopathic chronic intermediate uveitis [18]. Several immune alterations were previously described in experimental models of uveitis [20, 21]. According to our knowledge, there is still a lack of reports on the immune status of pars planitis patients, and the present work is the first study performed in humans. In this study, we observed a strongly pronounced imbalance in IL-17-producing (Th17) and T regulatory/suppressor (Treg and Tsup) cells in the circulation of patients. Moreover, our results strongly suggest IFN-*γ*-secreting (Th1) cell involvement in the pathogenesis and/or clinical course of pars planitis.

Although Th1 abundance in our cohort of patients was found to positively correlate with pars planitis duration (primarily in those with macular edema), worthy of note was their significant deficiency in the patients' circulation. The observed Th1 dysregulation in our study does not seem to be accidental, since a significantly decreased proportion of IFN-*γ*-producing cells was observed within both examined CD3+CD8- and CCR4-CXCR3+ subsets, which are known to be enriched for Th1 cells. Our knowledge on the role of IFN-*γ*-secreting cells in the pathogenesis of noninfectious uveitis derives from several experimental models, showing an increased Th1/Th2 ratio in acute disease [22], which may, in fact, result from a more pronounced defect in Th2 abundance. However, little is known about the exact role of Th1 in pars planitis in humans. Accordingly, there are only a few studies performed in patients with intermediate uveitis, showing conflicting results [23, 24]. Walscheid et al. [23] observed an increased Th1/Th2 ratio in active noninfectious uveitis. Meanwhile, Murphy et al. [24], in a study on PB CD4+ T cell expression of chemokine receptors assigned to Th1 (CXCR3) and Th2 (CCR4) lineages, found no difference between patients with active intermediate uveitis compared to both inactive disease and healthy donors [24]. Our current report showing a dysregulated Th1 compartment in pars planitis appears to be in line with the demonstration of the different roles of Th17 and Th1 cell subsets in, respectively, the induction and maintenance of inflammation in organ-specific autoimmune disorders [25]. There is a body of evidence showing that elicitation of an early Th17 response may promote and strengthen further recruitment of Th1 cells into the target tissue, thus leading to enforcement of the local inflammation and tissue injury over time. Therefore, our finding of Th1 systemic deficiency in pars planitis strongly points to the possibility of Th1 cell sequestration in the inflamed eye tissue. Nonetheless, we also found that Th1 abundance in the peripheral blood of patients systematically increased over time. More importantly, this observation concerned patients with macular edema only. At this stage, we cannot completely explain this phenomenon. However, regarding the thesis of RPE progressively losing its immunomodulatory properties in the course of pars planitis, we hypothesize that only a proportion of Th1 cells might be sufficiently converted into Treg cells for controlling the inflammation in the eye tissues. In consequence, accumulating Th1 cells are capable to sustain local inflammation and mediate tissue injury. On the other hand, this might also support Th1 redistribution between the eye tissue and circulation, which should also be considered primarily in patients with clinical complications, such as macular edema. Thus, pars planitis seems to be a Th1- rather than Th17-dependent entity, which is consistent with the former demonstration of Th1 association with organ-specific autoimmunity [26]. Therefore, our current study could shed light on the partial or lack of response to a human antibody to IL-17A (secukinumab) treatment in patients with noninfectious uveitis in a few clinical trials [27–29].

In the current study, we also observed a significantly higher percentage of proinflammatory IL-17-secreting (Th17) cells in the circulation of patients, while CD4+CD25hiCD127- Treg and CD8+CD28-FOXP3+ Tsup cell abundance was found to be diminished as compared to healthy controls. Similar findings were obtained in patients with uveitis related to systemic autoimmune inflammation or noninfectious uveitis [23, 30–32]. Although there are conflicting results on the changes in the number of Tregs in active disease [30, 31, 33, 34], most reports consistently emphasize their role in uveitis resolution and maintenance of the remission phase [30, 33, 35, 36]. The Th17/Treg imbalance found in our study suggests that all patients lose their immunomodulatory properties and can no longer maintain an equilibrium between proinflammatory and anti-inflammatory cells, including within educational gates (EGs). The idea of educational gates means that lymphocytes may migrate to specific tissues within privileged organs, where they learn how to react in site-specific immunological surroundings. The concept of EGs as the sites that are considered responsible for maintaining the immunological privilege was suggested by Shechter et al. in 2013 [37]. They described two immunological gates within the eye: the outer blood-retinal and blood-aqueous barrier. In both, the pigmented epithelium plays a crucial role, no matter whether it is the RPE, the ciliary body, or even the pigmented iris epithelium (IPE). The report by Azumi and Atherton [38] provided evidence of RPE involvement in the immunomodulation of inflammation even in an uninoculated eye after uniocular anterior chamber inoculation of herpes simplex virus. On the other hand, if the immune privilege is to be maintained by the EGs, an injury to a part of that gate (e.g., RPE) should produce an immune response within the whole eye and not only within the pars plana, as described in various animal models of uveitis [18]. However, none of the experimental models fully explains the changes that occur in the case of uveitis, including pars planitis.

In the case of B cell contribution to the immunopathogenesis of pars planitis, previous papers have described some local changes in enucleated eyes without correlating the outcomes with immunological findings in the circulation [1, 39]. The perivascular infiltrates were shown as consisting predominantly of T cells with a few B cells only. One should, however, note that the examination was performed in enucleated eyes at the final stage of the disease and by immunohistochemical assay, which makes any comparison difficult. Our present study is the first to evaluate the abundance of Breg cells, phenotyped as CD19+CD24hiCD38hi, in peripheral blood samples collected from pars planitis patients. We observed no statistically significant differences between patients and healthy controls, thus emphasizing the significance of alterations in the T cell repertoire in the pathogenesis of pars planitis. Further assessment of the cellular components of the immune system, primarily in intraocular fluids, may provide further insight into the mechanisms underlying pars planitis.

## 5. Conclusions

This is the first study to evaluate the distribution of pro- and anti-inflammatory subpopulations of lymphocytes in the peripheral blood of pars planitis patients. Our findings of the predominance of Th17 lymphocytes and dysregulated levels of both Treg and Th1 cells reflect a strong shift of the immune balance towards proinflammatory conditions and can be a foundation of patient-specific biologic treatment. In addition, the positive strong correlation of Th1 frequency with the duration of pars planitis observed in patients with macular edema suggests that circulating Th1 level may be a potential predictive factor of the most severe course of the disease.

## Figures and Tables

**Table 1:** Inclusion criteria for screening procedure as well as inclusion and exclusion criteria for study enrolment.

Inclusion criteria for screening	
Age > 18 years old Written informed consent Diagnosis of intermediate uveitis according to SUN criteria	

Inclusion criteria	Exclusion criteria
Written informed consent Established diagnosis of pars planitis: (i) Negative viral tests (for HSV-1 and HSV-2, VZV, HHV-7, CMV, Epstein-Barr virus, and HIV) (ii) Negative blood IgG and IgM levels for Borrelia burgdorferi, *Toxoplasma*, *Toxocara*, and Bartonella pertussis (iii) Negative QuantiFERON and skin reaction to tuberculin (Mantoux test with tuberculin RT23) (iv) Negative blood test for syphilis (Wassermann reaction and VDRL test) (v) No previous diagnosis of systemic autoimmune disease (vi) No pathologic findings in X-ray examination of the chest Patient's observation for at least the time necessary to establish the diagnosis of pars planitis including diagnostic tests and other specialist consultation (for at least 4 weeks)	Age < 18 or >70 years old No written consent Noncompliance with the medical monitoring (study protocol) Pregnancy and breast feeding Diabetes mellitus Hypertension Neoplastic disorder (diagnosed or suspected) Life-threatening diseases Oral or local steroid treatment in the last 4 weeks Oral or local immunomodulatory treatment in the last 6 months (such as methotrexate, azathioprine, mycophenolate mofetil, tacrolimus, cyclosporine, or cyclophosphamide) Any previous biological treatment (e.g., adalimumab, infliximab, rituximab, and anakinra)

## Data Availability

The datasets used and analyzed during the current study are all available from the corresponding author.

## Supplementary Materials

Supplementary Materials Table S1: summarized description of the pars planitis screening group. Table S2: detailed description of the pars planitis screening group. Table S3: summary of performed ophthalmological examinations including grading scales and equipment specification used. Figure S1: representative examples of cytometric analysis of IFN-*γ*-secreting cells in PB of pars planitis patients (PP) and healthy controls detected as (A) CD3+CD8- IFN-*γ*+ and (B) CCR4-CXCR3+ IFN-*γ*+ cells and shown as dot plots. The gating strategy was performed as follows: PB mononuclear cells were gated on the basis of forward- and side-scatter properties (FSC vs. SSC), and shown as R1. The R2 gate (made in the SSC and CD3 or CCR4 windows) marked CD3+ and CCR4- cells, respectively. Numbers on dot plots represent the frequency of IFN-*γ*-secreting cells within CD3+CD8- (A) and CCR4-CXCR3+ cells (B) of PP patients and healthy controls. The percentages of cells expressing IFN-*γ* were determined using isotype control IgG. Figure S2: representative examples of cytometric analysis of IL-17-secreting cells in PB of pars planitis patients (PP) and healthy controls detected as (A) CD3+CD8-IL-17+ and (B) CCR4+CCR6+IL-17+ cells are shown as dot plots. The gating strategy is also shown: R1 represents the gated PB mononuclear cells in the SSC and FSC window. Then, lymphocytes were gated in R2 on the basis of CD3 or CCR4 fluorescence and SSC scatter properties, demonstrating CD3+ or CCR4+ cells, respectively. Numbers on dot plots represent the frequency of IL-17A-secreting cells within CD3+CD8- (A) and CCR4+CCR6+ cells (B) of PP patients and healthy controls. The percentages of cells expressing IL-17 were determined using isotype control IgG. Figure S3: representative examples of cytometric analysis of regulatory/suppressor cells in PB of pars planitis patients (PP) and healthy controls detected as (A) CD4+CD25hiCD127- T regulatory cells, (B) CD8+CD28-FOXP3+ T suppressor cells, and (C) CD19+CD24hiCD38hi B regulatory cells are shown as dot plots. The gating strategy is also shown: R1 gate exhibits PB mononuclear cells according to FSC and SSC scatter properties. R2 gate allows designation of CD4+, CD8+, or CD19+ lymphocytes in the fluorescence and SSC scatter window. Numbers on dot plots represent the Treg, Tsup, or Breg cell frequencies in PB of PP patients and healthy controls. The percentages of cells expressing studied antigens were determined using isotype control IgG.
