# Supplementary material for: Imbalance in PB IL-17-Secreting and Regulatory Cells in Pars Planitis Is Associated with Dysregulation of IFN-γ-Secreting Cells, Especially in Patients with Clinical Complications
Source: Mediators Inflamm. 2020 Jul 31;2020:9175083. doi: 10.1155/2020/9175083 (PMC7415078; doi:10.1155/2020/9175083)
Supplement: Supplementary Materials — Table S1: summarized description of the pars planitis screening group. Table S2: detailed description of the pars planitis screening group. Table S3: summary of performed ophthalmological examinations including grading scales and equipment specification used. Figure S1: representative examples of cytometric analysis of IFN-γ-secreting cells in PB of pars planitis patients (PP) and healthy controls detected as (A) CD3+CD8- IFN-γ+ and (B) CCR4-CXCR3+ IFN-γ+ cells and shown as dot plots. The gating strategy was performed as follows: PB mononuclear cells were gated on the basis of forward- and side-scatter properties (FSC vs. SSC), and shown as R1. The R2 gate (made in the SSC and CD3 or CCR4 windows) marked CD3+ and CCR4- cells, respectively. Numbers on dot plots represent the frequency of IFN-γ-secreting cells within CD3+CD8- (A) and CCR4-CXCR3+ cells (B) of PP patients and healthy controls. The percentages of cells expressing IFN-γ were determined using isotype control IgG. Figure S2: representative examples of cytometric analysis of IL-17-secreting cells in PB of pars planitis patients (PP) and healthy controls detected as (A) CD3+CD8-IL-17+ and (B) CCR4+CCR6+IL-17+ cells are shown as dot plots. The gating strategy is also shown: R1 represents the gated PB mononuclear cells in the SSC and FSC window. Then, lymphocytes were gated in R2 on the basis of CD3 or CCR4 fluorescence and SSC scatter properties, demonstrating CD3+ or CCR4+ cells, respectively. Numbers on dot plots represent the frequency of IL-17A-secreting cells within CD3+CD8- (A) and CCR4+CCR6+ cells (B) of PP patients and healthy controls. The percentages of cells expressing IL-17 were determined using isotype control IgG. Figure S3: representative examples of cytometric analysis of regulatory/suppressor cells in PB of pars planitis patients (PP) and healthy controls detected as (A) CD4+CD25hiCD127- T regulatory cells, (B) CD8+CD28-FOXP3+ T suppressor cells, and (C) CD19+CD24hiCD38hi B regulatory cells ar [file 9175083.f1.doc]

**Table S1 Summarized description of pars planitis screening group.**

| **Test** | **Pars planitis screening (n=29)**** | |
| --- | --- | --- |
|  | **Positive** | **Negative** |
| **Borrelia burgdorferi IgG** | 4 | 25 |
| **Borrelia burgdorferi IgM and IgG** | 1 | 28 |
| **Quantiferon/Mantoux test** | 3 | 26 |
| **Viral multitest** | 2 | 27 |
| **Suspected systemic disease** | 2 | 27 |
| **Tuberculosis (vitreal samples)*** | 1 | 28 |

*Obtained after screening due to rapid bilateral exacerbation of symptoms; **One patient withdrew their consent after screening

**Table S2** Detailed description of pars planitis screening group.

| **Patient** | **Borrelia burgdorferi** | | **Quantiferon/Mantoux** | **Viral multitest** | **Suspicion of systemic disease** | **Withdrawal of written consent** | **Lost after screening** | **Cause** |
| --- | --- | --- | --- | --- | --- | --- | --- | --- |
|  | **IgG** | **IgG and IgM** |  |  |  |  |  |  |
| **1** |  |  |  |  |  |  | yes | Tuberculosis in vitreal samples (PCR) |
| **2** | yes |  |  |  |  |  |  |  |
| **3** |  |  |  |  |  |  |  |  |
| **4** |  |  |  |  |  |  |  |  |
| **5** |  |  |  |  |  |  |  |  |
| **6** | yes |  |  |  |  |  |  |  |
| **7** |  |  |  |  |  |  |  |  |
| **8** |  |  |  |  |  |  |  |  |
| **9** |  | yes |  |  |  |  |  |  |
| **10** |  |  |  |  |  |  |  |  |
| **11** |  |  |  |  |  |  |  |  |
| **12** |  |  | yes |  |  |  |  |  |
| **13** |  |  |  |  |  |  |  |  |
| **14** |  |  |  |  |  |  |  |  |
| **15** | yes |  |  |  |  |  |  |  |
| **16** |  |  |  |  |  |  |  |  |
| **17** |  |  |  | yes |  |  |  |  |
| **18** | yes |  |  |  |  |  |  |  |
| **19** |  |  |  |  |  |  |  |  |
| **20** |  |  |  |  |  |  |  |  |
| **21** |  |  | yes |  |  |  |  |  |
| **22** |  |  |  |  |  |  |  |  |
| **23** |  |  |  |  | yes |  |  |  |
| **24** |  |  |  |  |  |  |  |  |
| **25** |  |  |  | yes |  |  |  |  |
| **26** |  |  |  |  |  |  |  |  |
| **27** |  |  |  |  |  | yes |  |  |
| **28** |  |  |  |  |  |  |  |  |
| **29** |  |  | yes |  |  |  |  |  |

**Table S3 Summary of performed ophthalmological examinations including grading scales and equipment specification used.**

| **List of Performed Ophthalmological Examinations** |
| --- |
| 1. BCVA tested using ETDRS charts (different charts for each eye) from the distance of 4 m 2. NV tested using Snellen charts from the distance of 25 cm 3. Evaluation of anterior segment of the eye (slit lamp examination) including assessment of the inflammation (Anterior chamber cell count, Anterior chamber flare count, Vitreous haze scale according to SUN) 4. IOP measurement performed with GAT 5. Application of 1 drop of 10% Neosynephrin and 1% Tropicamid 6. Assessment of the lens opacity and vitreal inflammation 7. Fundoscopy 8. OCT examination of macula and submacular choroid |

BCVA – best-corrected visual acuity, IOP – intraocular pressure, GAT – Goldmann applanation tonometry


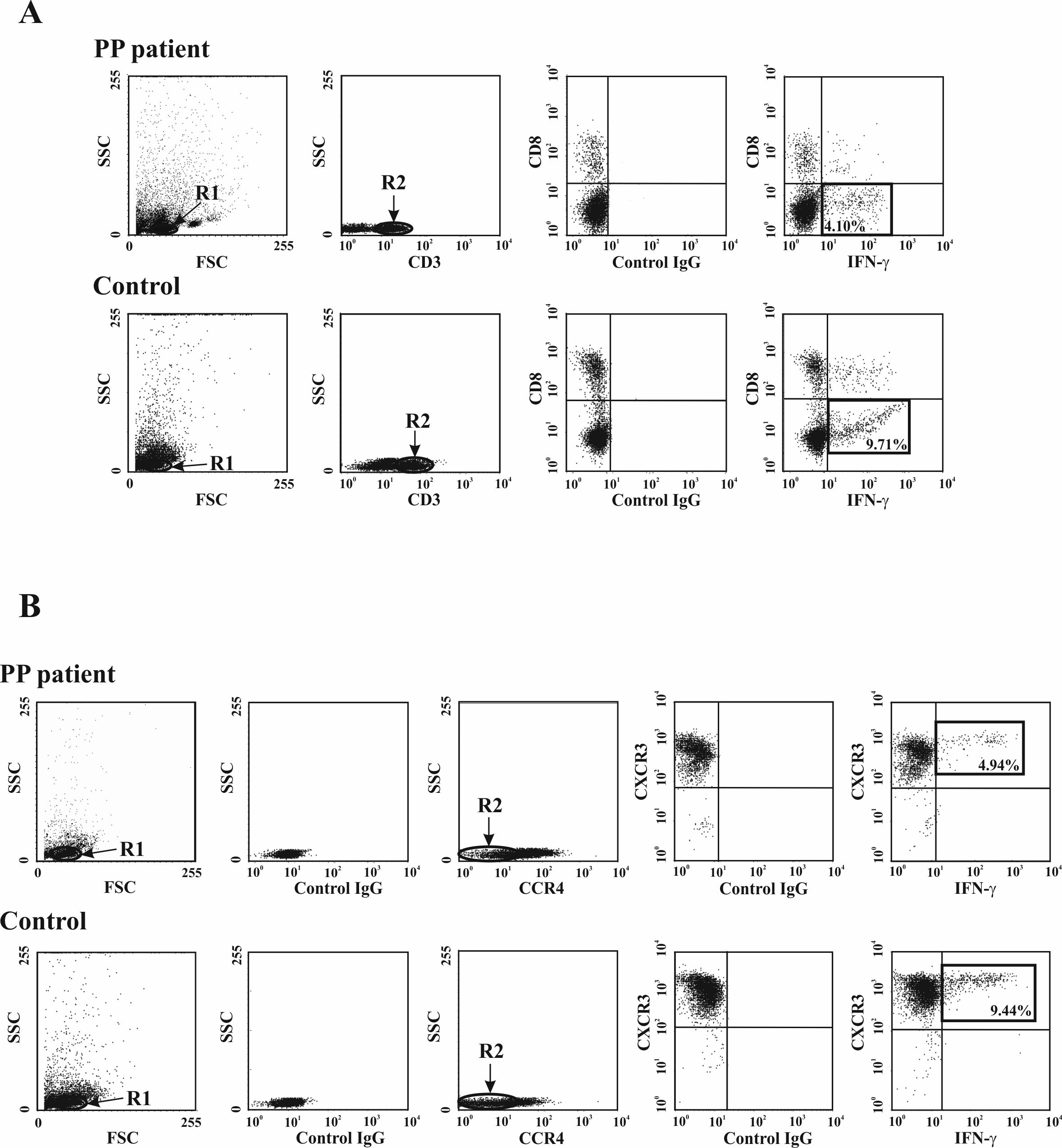


**Figure S1** Representative examples of cytometric analysis of IFN-γ-secreting cells in PB of pars planitis patients (PP) and healthy controls detected as: **A**) CD3+CD8- IFN-γ+ and **B**) CCR4-CXCR3+ IFN-γ+ cells and shown as dot plots. The gating strategy ~~was also shown~~ was performed as follows: PB mononuclear cells were gated on the basis of forward- and side-scatter properties (FSC vs. SSC), and shown as R1. The R2 gate (made in the SSC and CD3 or CCR4 windows) marked CD3+ and CCR4- cells, respectively. Numbers on dot plots represent the frequency of IFN-γ-secreting cells within CD3+CD8- (A) and CCR4-CXCR3+ cells (B) of PP patients and healthy controls. The percentages of cells expressing IFN-γ were determined using isotype control IgG.


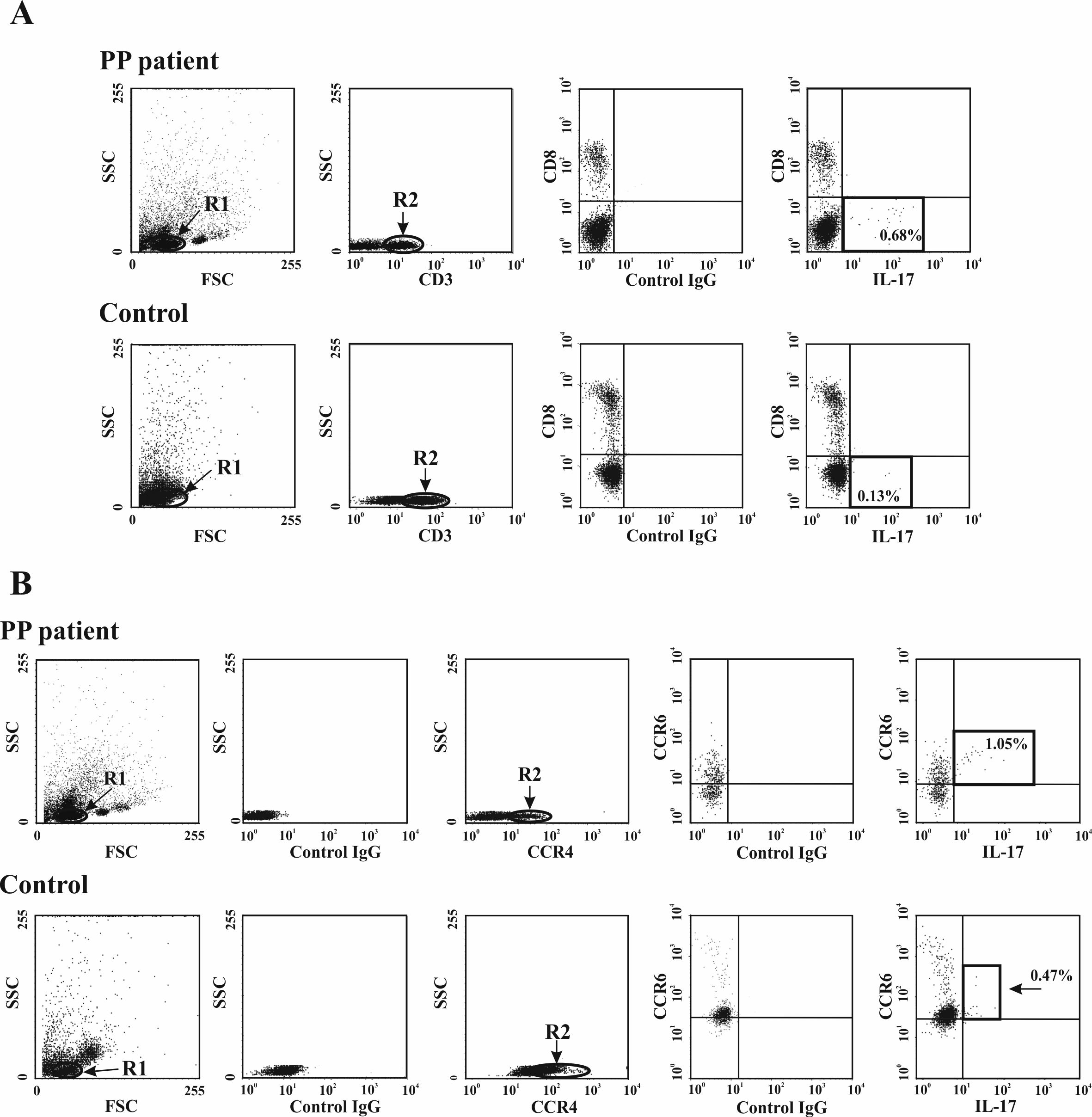


**Figure S2** Representative examples of cytometric analysis of IL-17-secreting cells in PB of pars planitis patients (PP) and healthy controls detected as: **A**) CD3+CD8-IL-17+ and **B**) CCR4+CCR6+IL-17+ cells are shown as dot plots. The gating strategy is also shown: R1 represents the gated PB mononuclear cells in the SSC and FSC window. Then lymphocytes were gated in R2 on the basis of CD3 or CCR4 fluorescence and SSC scatter properties, demonstrating CD3+ or CCR4+ cells, respectively. Numbers on dot plots represent the frequency of IL-17A-secreting cells within CD3+CD8- (A) and CCR4+CCR6+ cells (B) of PP patients and healthy controls. The percentages of cells expressing IL-17 were determined using isotype control IgG.


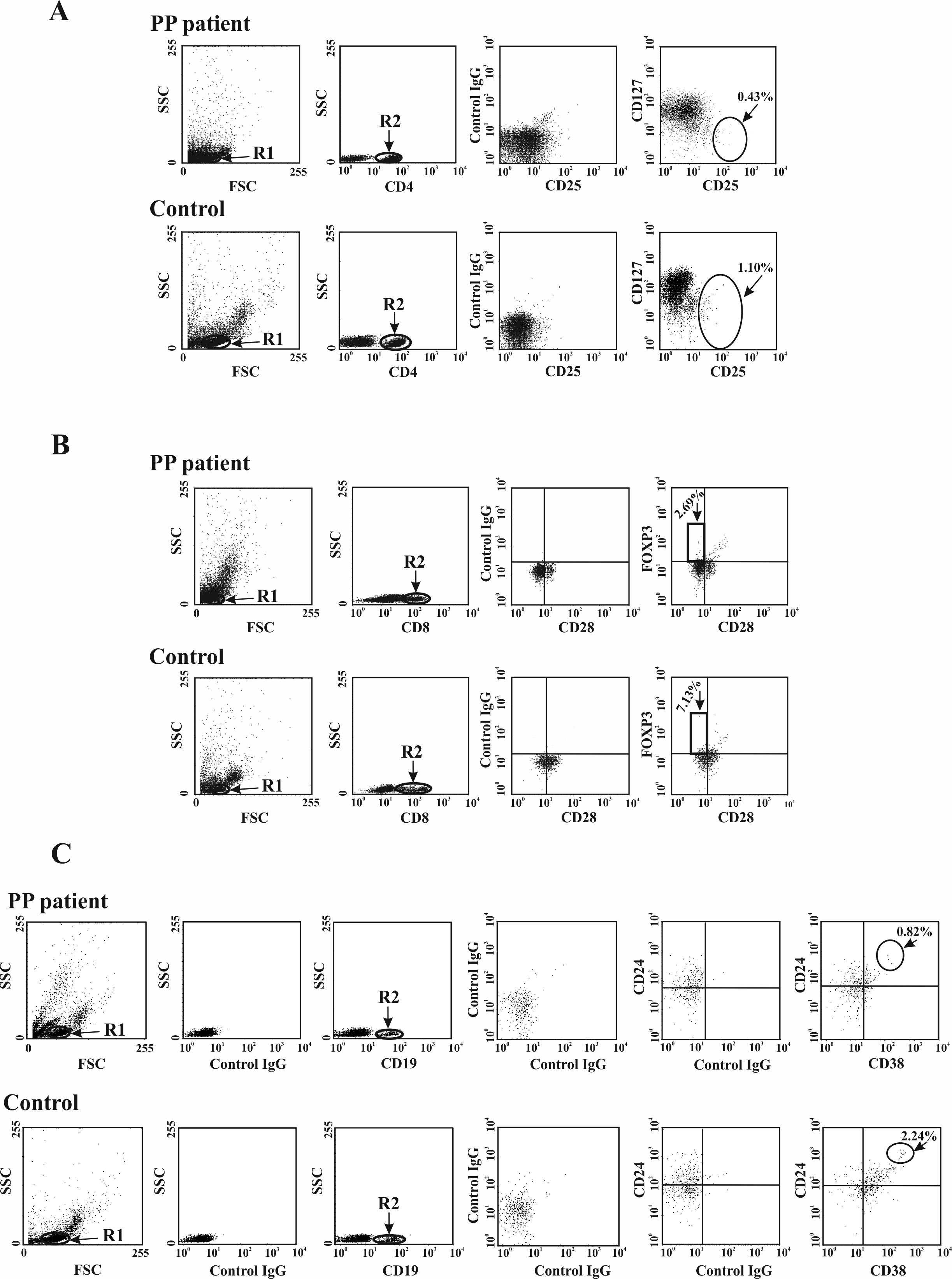


**Figure S3** Representative examples of cytometric analysis of regulatory/suppressor cells in PB of pars planitis patients (PP) and healthy controls detected as: **A**) CD4+CD25hiCD127- T regulatory cells, **B**) CD8+CD28-FOXP3+ T suppressor cells , and **C**) CD19+CD24hiCD38hi B regulatory cells are shown as dot plots. The gating strategy is also shown: R1 gate exhibits PB mononuclear cells according to FSC and SSC scatter properties. R2 gate allows designation of CD4+, CD8+, or CD19+ lymphocytes in the fluorescence and SSC scatter window. Numbers on dot plots represent the Treg, Tsup, or Breg cell frequencies in PB of PP patients and healthy controls. The percentages of cells expressing studied antigens were determined using isotype control IgG.
